# Supplementary material for: Multi-scale chromatin state annotation using a hierarchical hidden Markov model
Source: Nat Commun. 2017 Apr 7;8:15011. doi: 10.1038/ncomms15011 (PMC5385569; doi:10.1038/ncomms15011)
Supplement: Supplementary Information — Supplementary figures. [file ncomms15011-s1.pdf]

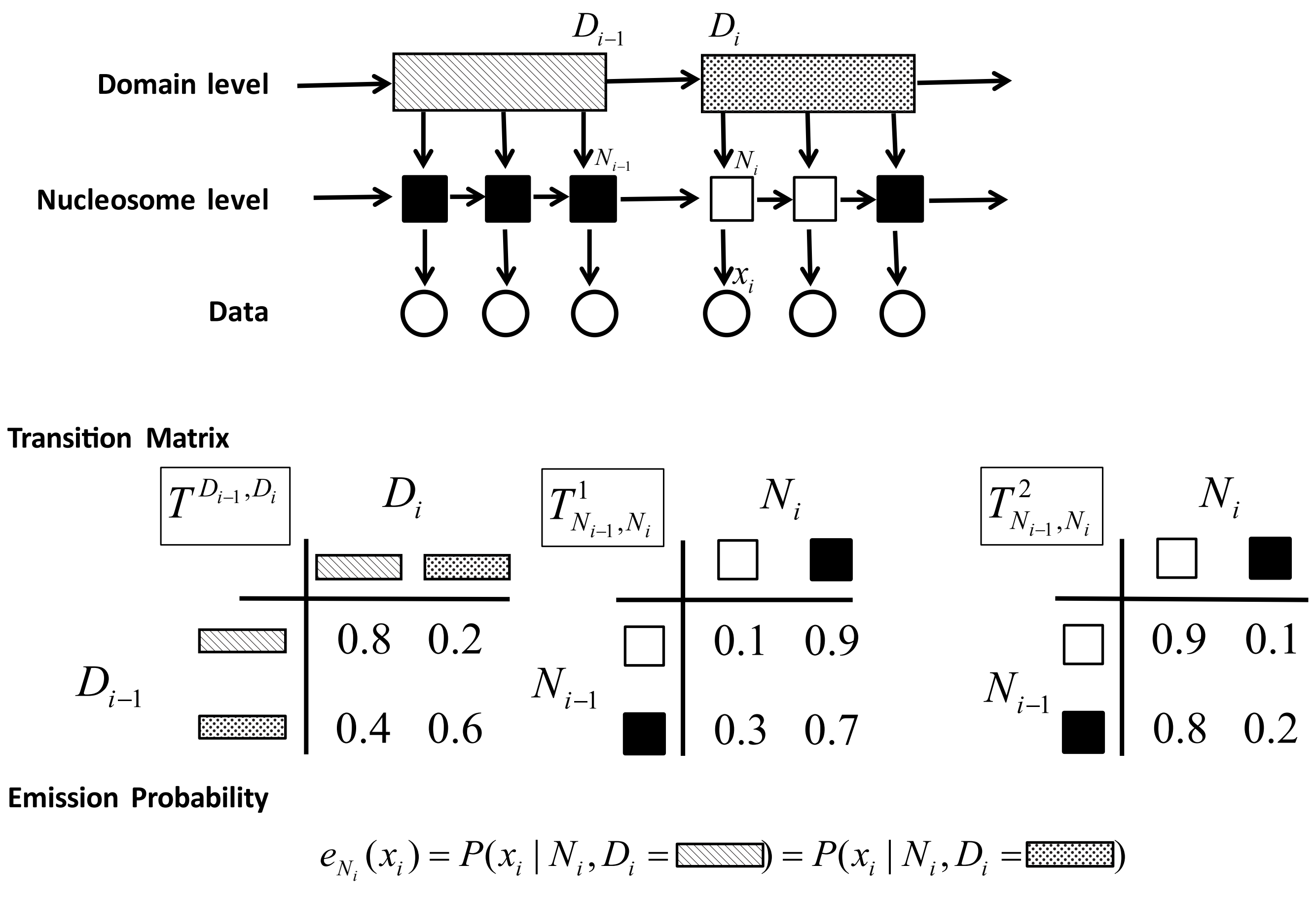

Supplementary Figure 1. A schematic overview of the mathematical properties of diHMM

diHMM contains two levels of hidden states, called domain- and nucleosome-level states, respectively. Each domain-level state is characterized by its own pattern of nucleosome-level state organizations. In the simplified example illustrated here, domain-level state 2 is enriched with white nucleosome-level states, whereas domain-level state 1 is enriched with black nucleosome-level states. The transition matrix reflects the probability of switching from one nucleosome-level state to another and is dependent on the underlying domain-level state. Domain transitions can only occur at the boundary of each 20 bin block. However, the emission probability is conditionally independent of the domain-level state given the nucleosome-level state. This decoupling of chromatin states into two levels facilitates the identification of a large number of combinatorial patterns using a relatively small number of hidden states.

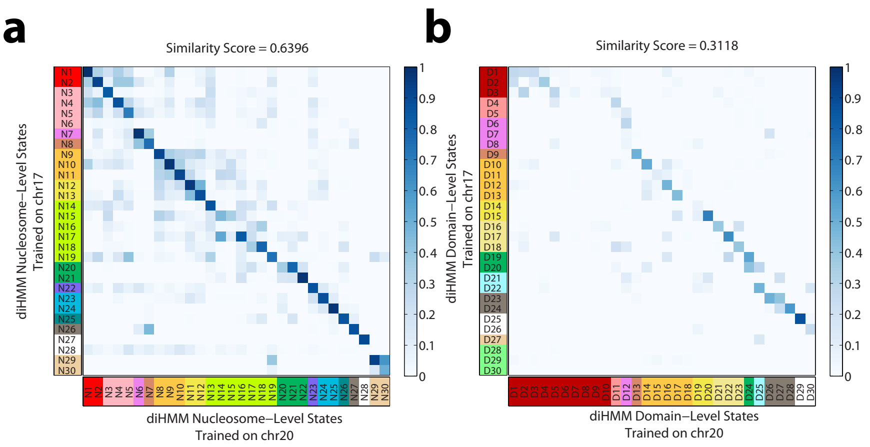

Supplementary Figure 2. Comparison between diHMM states obtained using models trained on chr17 and chr20

Heatmaps showing correlation matrices for the comparison between diHMM states obtained using models trained on chr17 and chr20. Shown are comparisons of **(a)** diHMM nucleosome-level states, and **(b)** diHMM domains-level states. Similarity scores are displayed above each heatmap.

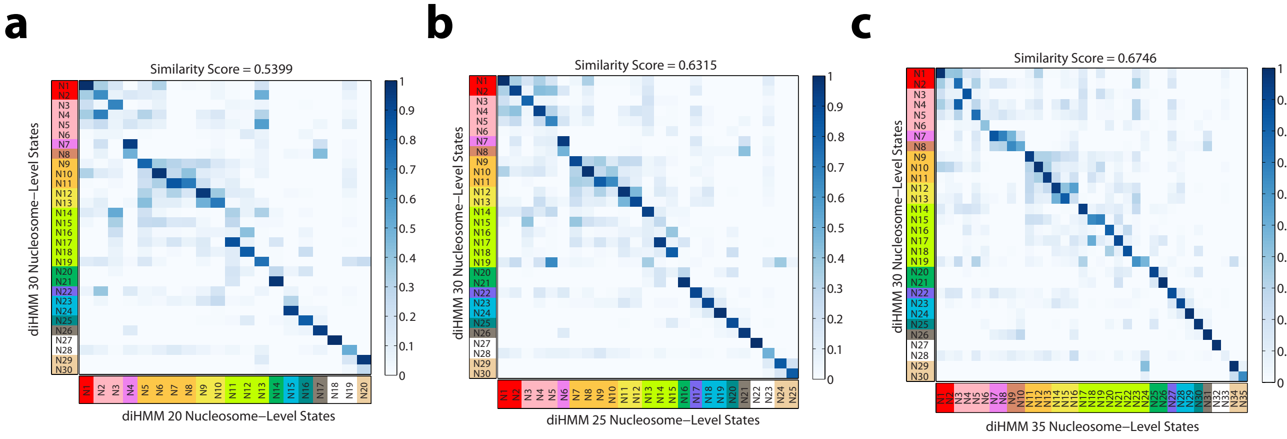

Supplementary Figure 3. Comparison between diHMM nucleosome-level states obtained using models with varying number of nucleosome-level states

Heatmaps showing correlation matrices for the comparison between diHMM nucleosome-level states obtained using models with 30 nucleosome-level states and **(a)** 20, **(b)** 25, and **(c)** 35 nucleosome-level states. Similarity scores are displayed above each heatmap.

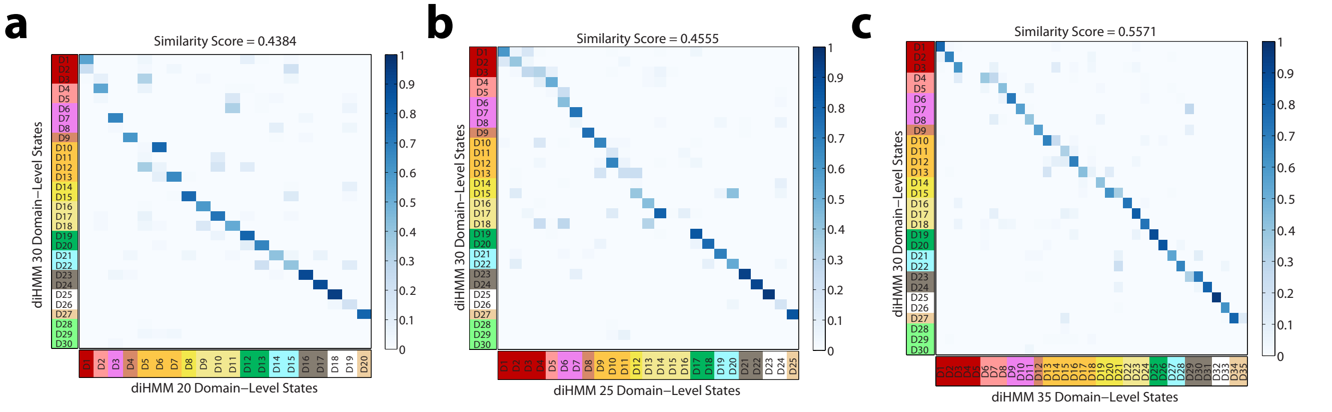

Supplementary Figure 4. Comparison between diHMM domain-level states obtained using models with varying number of domain-level states

Heatmaps showing correlation matrices for the comparison between diHMM domain-level states obtained using models with 30 domain-level states and **(a)** 20, **(b)** 25, and **(c)** 35 domain-level states. Similarity scores are displayed above each heatmap.

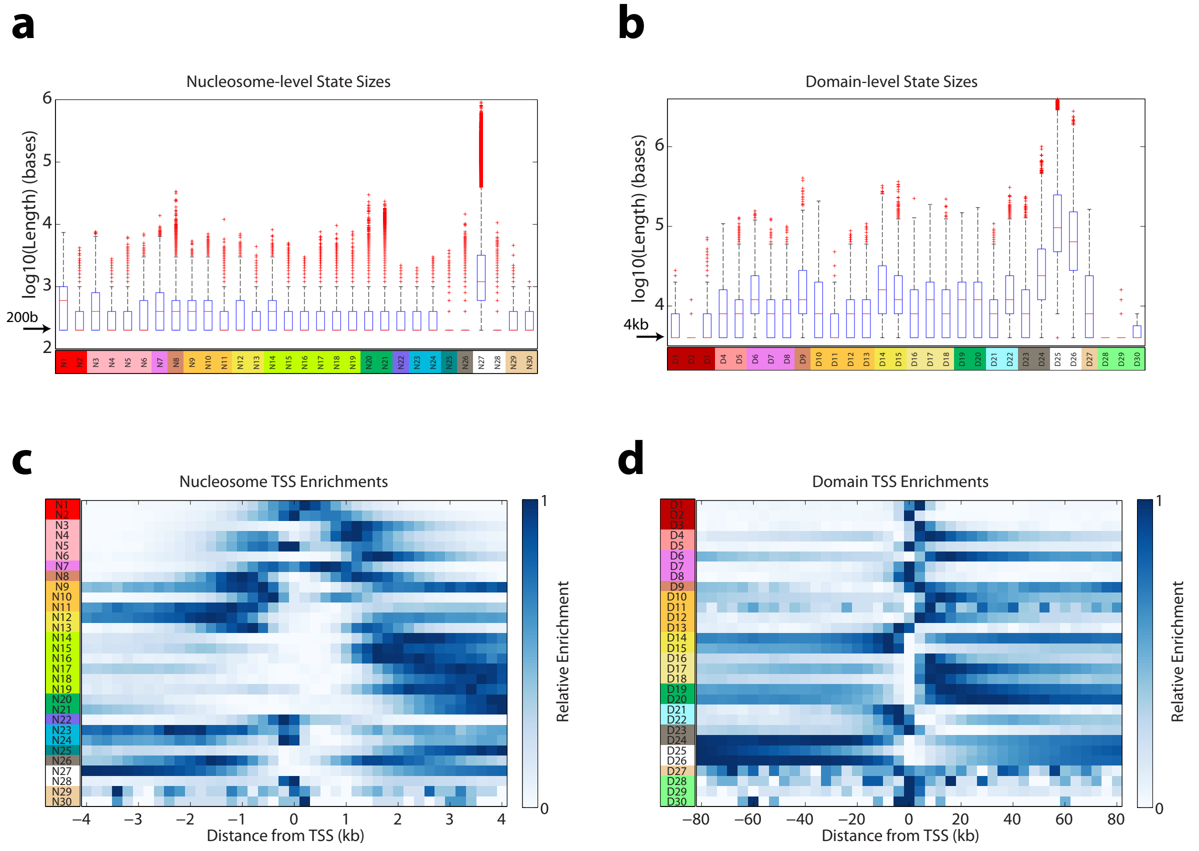

Supplementary Figure 5. Spatial distribution of the nucleosome- and domain-level diHMM states

Quartile box plots of log10 of all nucleosome-level (**a**), or domain-level (**b**) state sizes. Box plot whiskers extend to 1.5 times the interquartile range. Heatmaps show relative enrichment around TSS for (**c**) nucleosome-level states and (**d**) domain-level states. The scale varies linearly between 0 and 1.

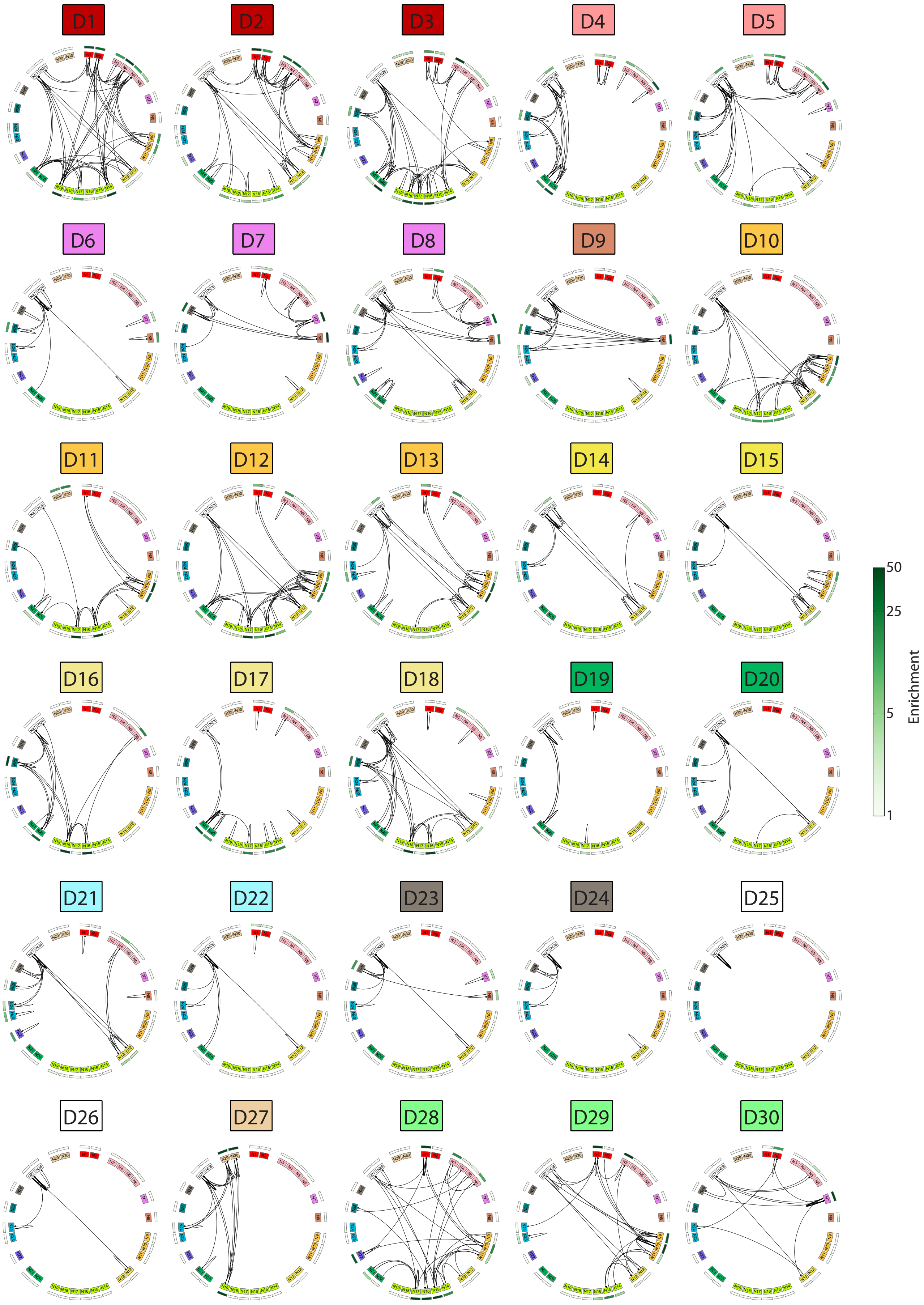

Supplementary Figure 6. Transitional properties of domain-level states

Circos plots showing most frequent nucleosome-level state transitions in each domain. Only transitions for which $T^{\nu}_{N,j\,k}$ has a minimum value of 0.01 are shown. The thickness of the link indicates how often each transition is used. Outer ring displays nucleosome-level enrichments in each domain as in **Fig. 2d**. The scale varies logarithmically between 1 (white) and 50 (dark green).

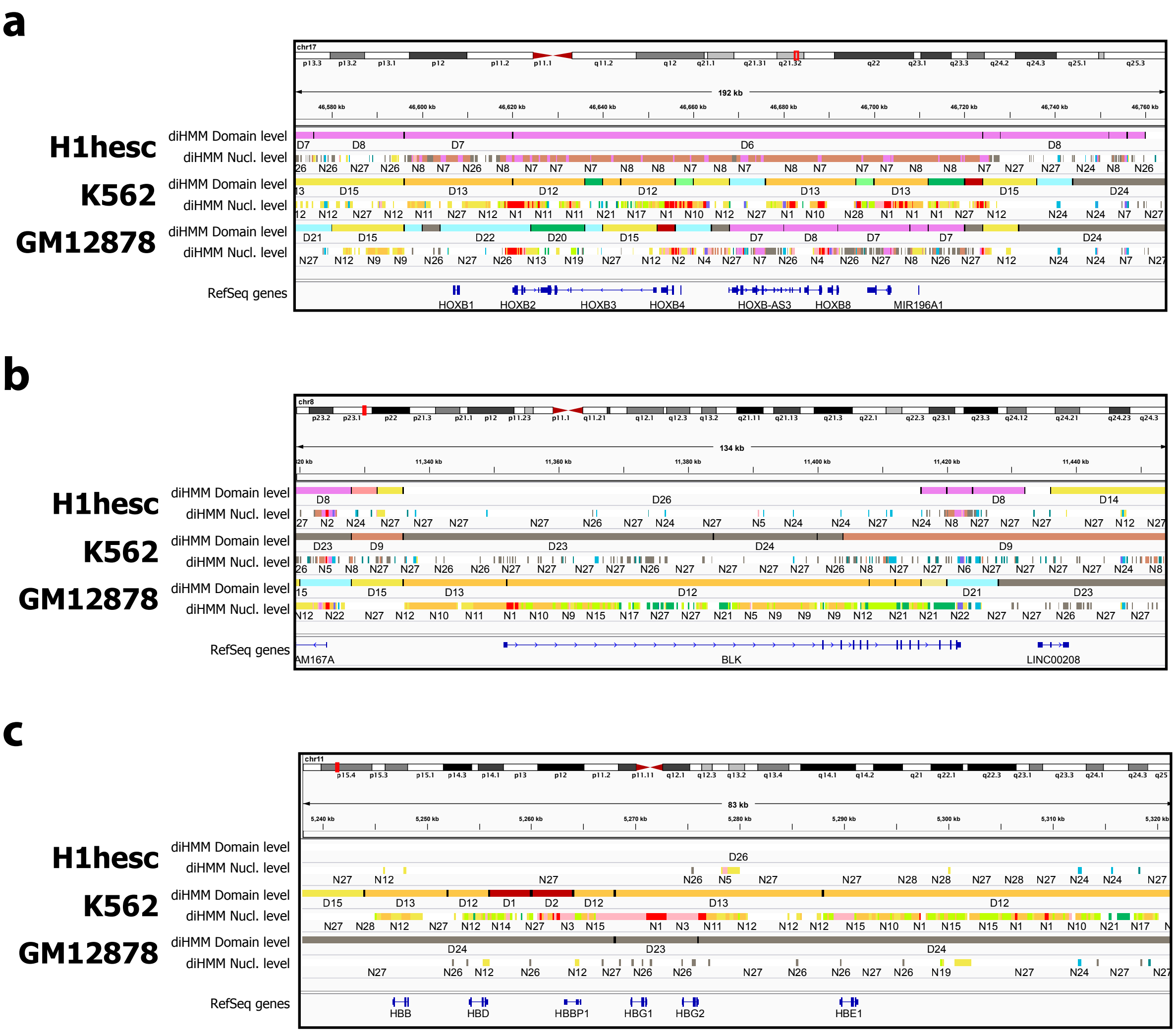

Supplementary Figure 7. Variation of diHMM states across three ENCODE Tier 1 cell lines

Genome tracks displaying diHMM state calls in H1, K562 and GM12878 cells for domain- and nucleosome-level states in three different regions. **(a)** shows the HOXB cluster region in chromosome 17 The most notable change is transition from a majority of Bivalent Promoter domains in H1, to Super-Enhancer domains in K562, to a mixture of domains in GM12878. In **(b)** the region around the gene BLK in chromosome 8 is shown. This region is characterized by the presence of Super-Enhancer domains in GM12878. In H1 cells the region is mostly absent of marks and in K562 the region is mostly repressed. In **(c)** we show the human β-globin locus in chromosome 11, with Super-Enhancers present only in K562 cells.

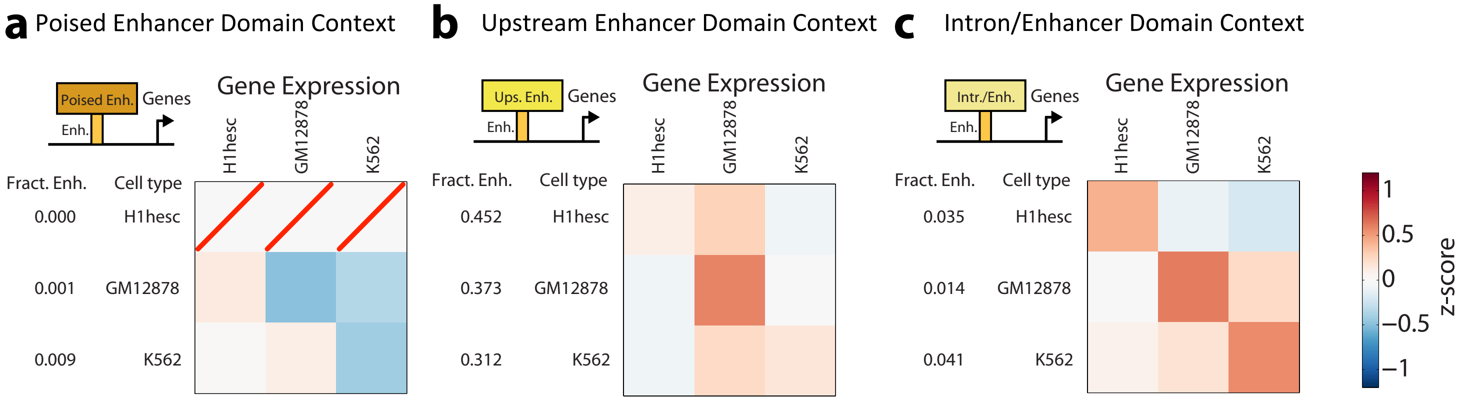

Supplementary Figure 8. Context-specific functionality of diHMM nucleosome-level enhancer states

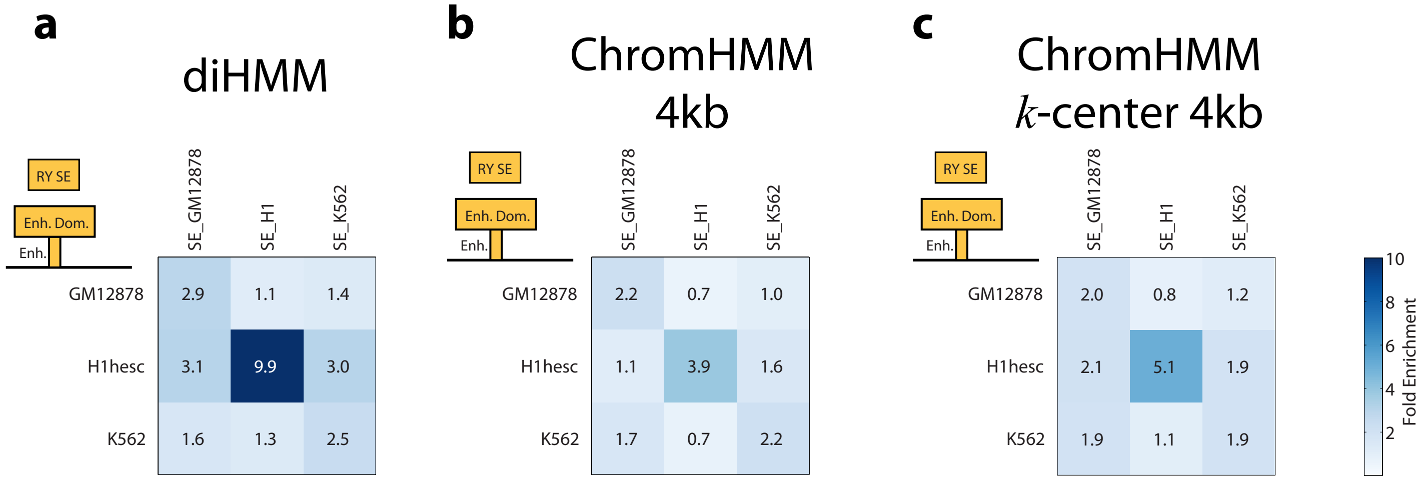

Supplementary Figure 9. Comparison between diHMM and two versions of ChromHMM-derived domains in association with super-enhancers

Heatmaps show the fold enrichment in Young lab super-enhancers[23] for enhancers in **(a)** diHMM Super-Enhancer domains, **(b)** ChromHMM 4kb states, and **(c)** *k*-center 4kb states.

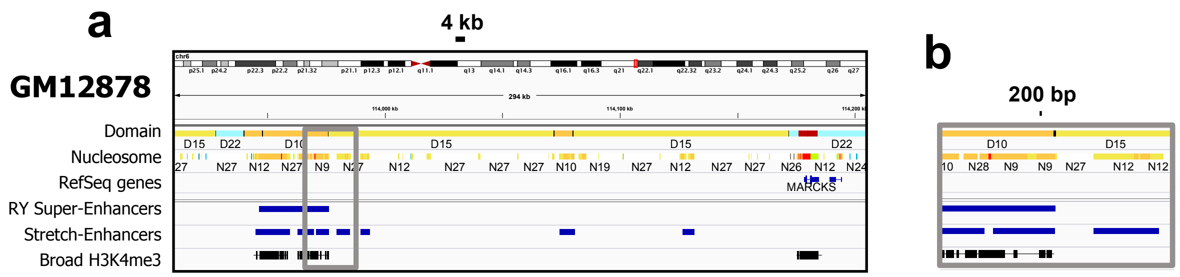

Supplementary Figure 10. Comparison between Super-Enhancer domains in diHMM and various domain structures reported in the literature

(a), genome tracks displaying diHMM annotation of domain- and nucleosome-level states in GM12878 cells. The area near MARCKS (a gene associated with chronic lymphocytic leukemia in B cells[4]) has several large Upstream Enhancer domains (D15) and a Super-enhancer domain (D10). Bottom tracks display super-enhancers[23], stretch enhancers[22] and buffer domains[24]. Gray box is expanded in **(b)** and shows a region of 20 kb.

a

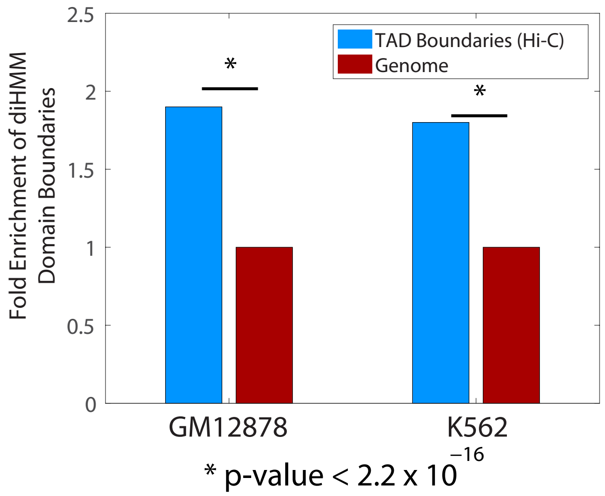

b

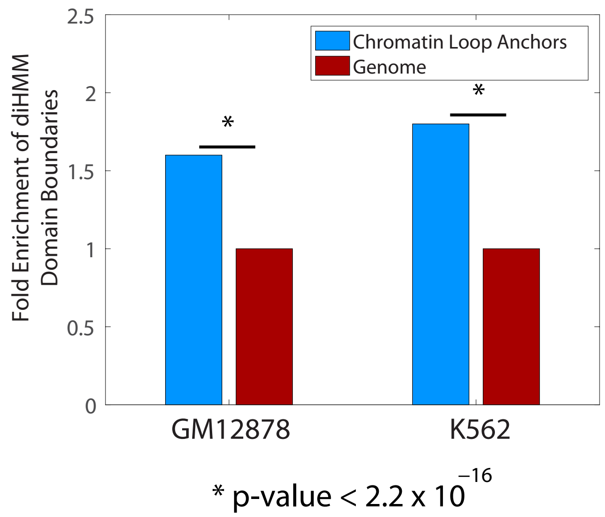

c

HUBS

Super-Enhancer Domains	GM12878	K562
GM12878	1.343	1.145
K562	1.112	1.153
H1hesc	1.522	1.408

All tests have p-value $< 2.2 \times 10^{-16}$

Supplementary Figure 11. diHMM domains and comparison with several Hi-C features

(a) shown are the fold enrichments of Topologically Associated Domains (TAD) in diHMM domain boundaries vs the whole genome. (**b**) shown are the fold enrichments of chromatin loop anchors in diHMM boundaries vs the whole genome. **(c)**, enrichment of Hi-C interaction hubs[25] in GM12878 and K562 (columns) for nucleosome-level enhancers states in diHMM (N9–N13) in Super-Enhancer domains (D10–D13) versus in the rest of the domains. All statistical tests in **(a-c)** are done using Fisher's exact test

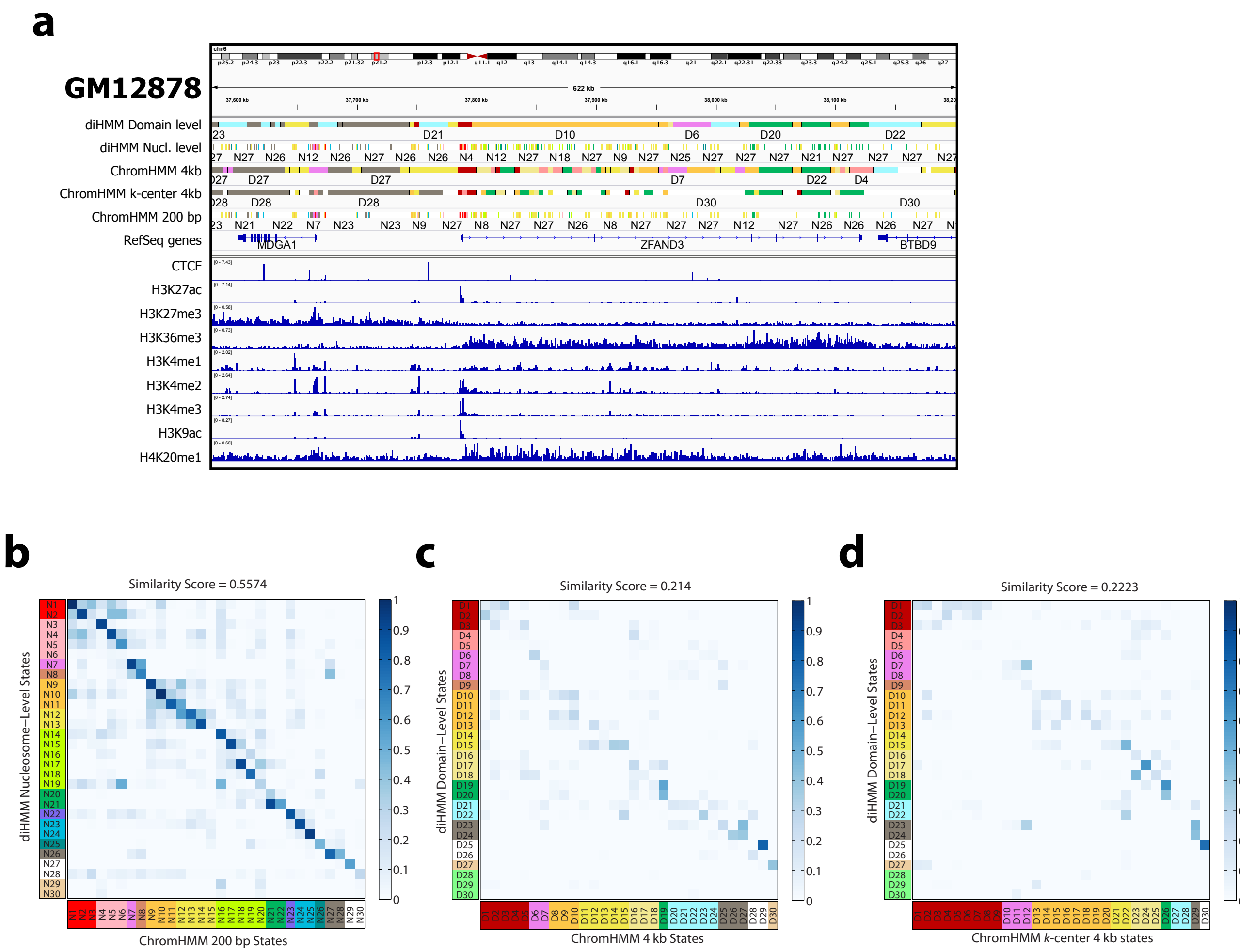

Supplementary Figure 12. Relationship between diHMM and ChromHMM

(**a**) Genome tracks in GM12878 showing nucleosome- and domain-level state calls for diHMM, and the counterpart obtained from alternative methods: ChromHMM 200 bp, ChromHMM 4kb, and *k*-center 4kb. Heatmaps showing correlation matrices for the comparison between (**b)** diHMM nucleosome-level states and ChromHMM 200 bp states, (**c)** diHMM domain-level states and ChromHMM 4kb states, and (**d)** diHMM domain-level states and *k*-center 4kb states. Similarity scores (see Methods) are displayed above each heatmap.

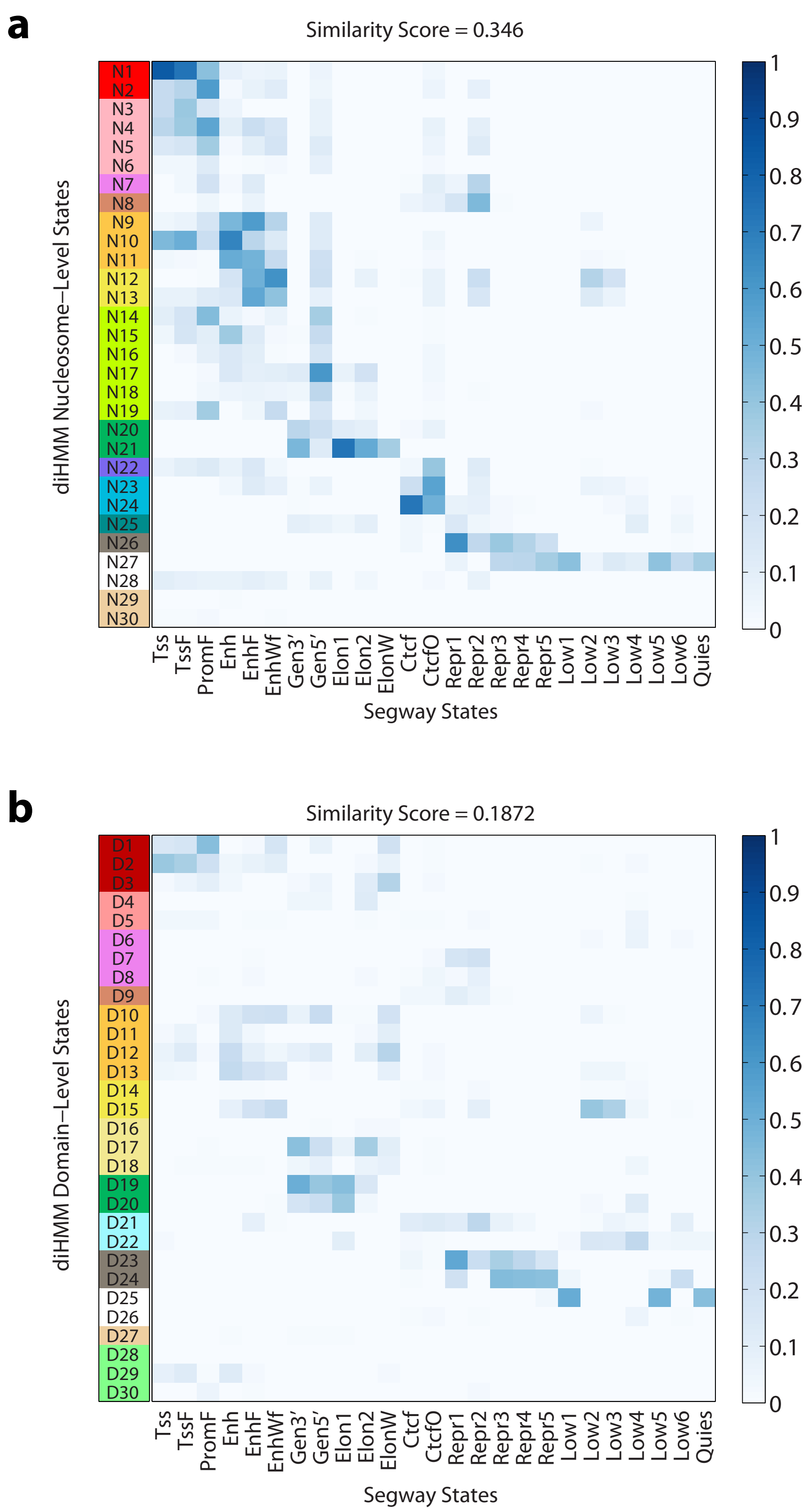

Supplementary Figure 13. Comparison between diHMM states and Segway states

Heatmaps showing correlation matrices for the comparison between Segway states[10] and **(a)** diHMM nucleosome-level states, and **(b)** diHMM domains-level states. Similarity scores (see Methods) are displayed above each heatmap and show that Segway states are more similar to diHMM nucleosome-level states.

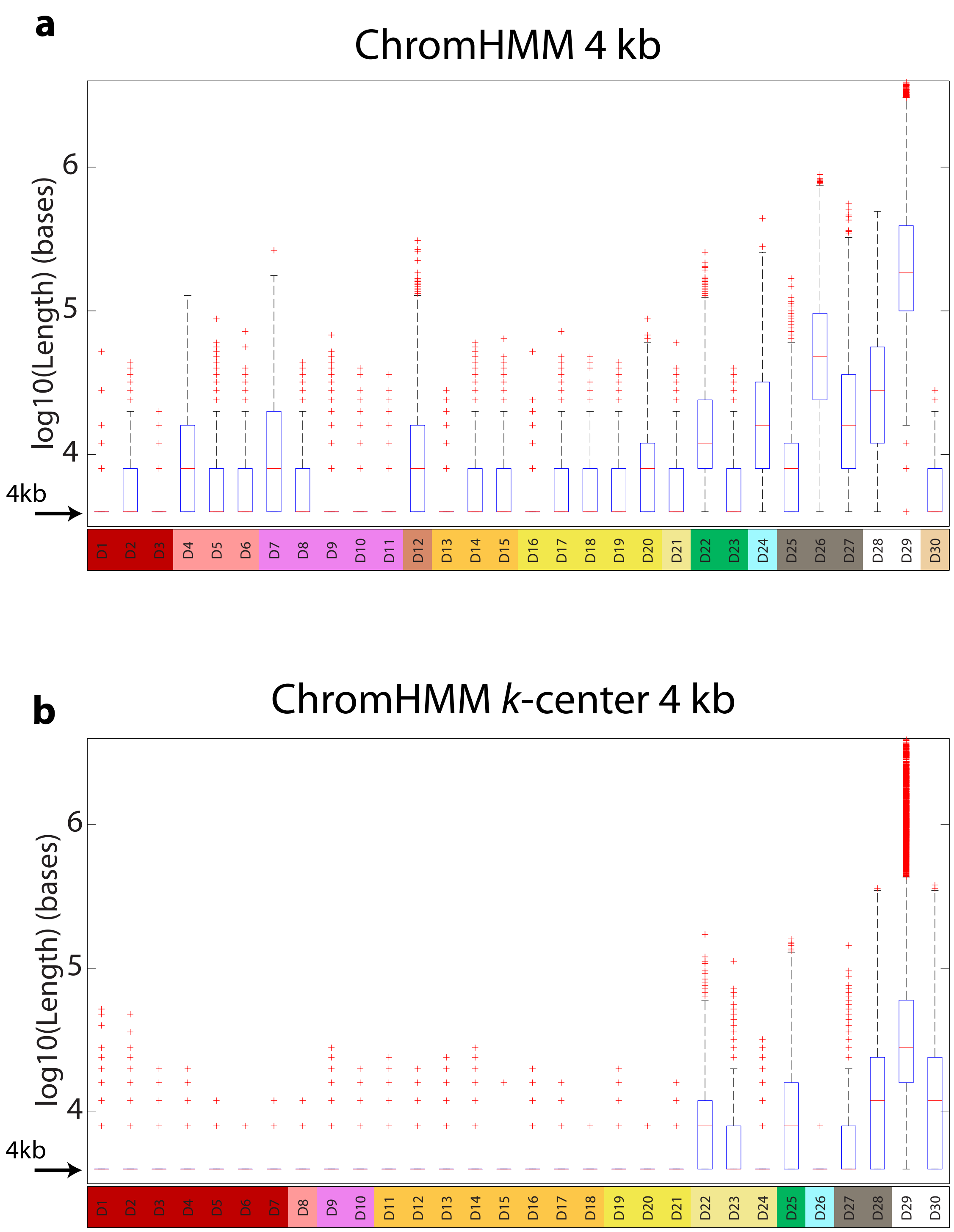

Supplementary Figure 14. Comparison between chromatin state sizes for **(a)** ChromHMM 4kb, and **(b)** ChromHMM *k*-center 4kb models

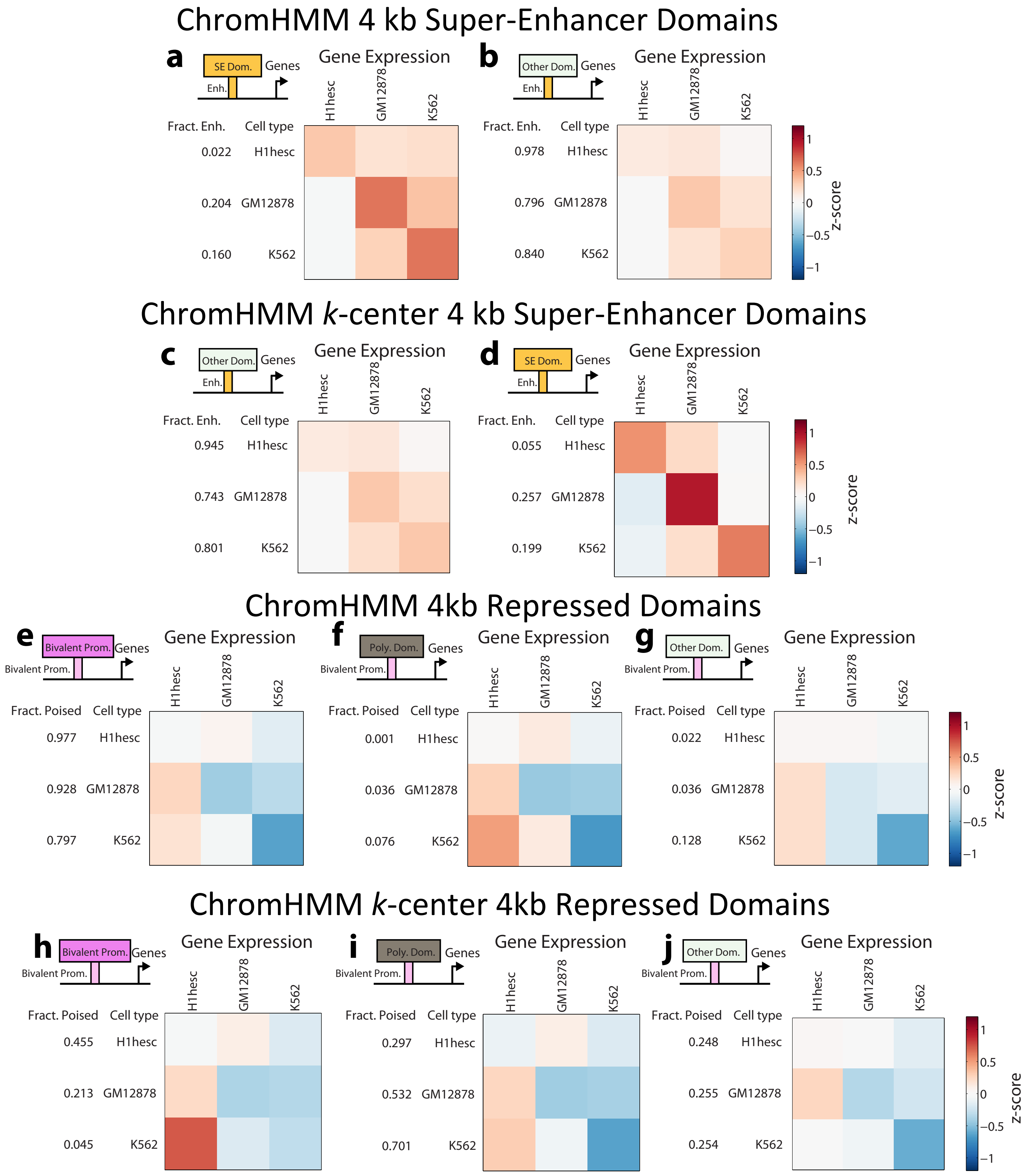

Supplementary Figure 15. Context-specific functionality of nucleosome-level states based on ChromHMM-derived domains

Similar to **Fig. 3** but for ChromHMM 4kb and k-center 4kb states. (**a**–**d**), average expression of genes mapped to enhancers in different domain contexts. (**e**–**j**), average expression of genes mapped to Bivalent Promoter nucleosome state N6 in different domain contexts.

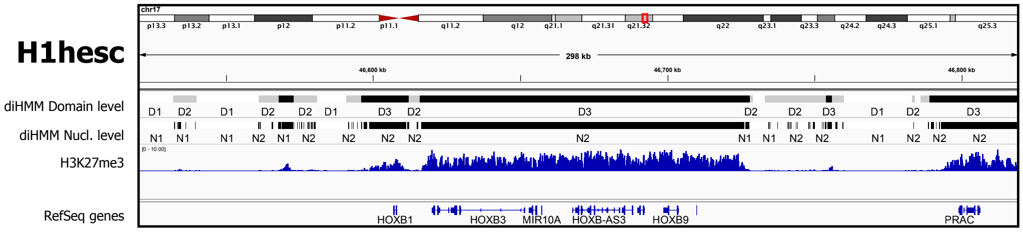

Supplementary Figure 16. Multi-scale chromatin state annotation from a single histone mark

Genome tracks displaying diHMM state calls, based on H3K27me3 alone, in H1 cells for domain- and nucleosome-level states in the HOXB cluster region in chromosome 17. The model contains three domain- and two nucleosome-level states. The two nucleosome-level states represent local presence or absence of H3K27me3, whereas the three domain-level states correspond to broad domain, desert, and their boundary.
